# Supplementary material for: A qualitative study to assess perceptions, barriers, and motivators supporting smokeless tobacco cessation in the US fire service
Source: PLoS One. 2021 May 11;16(5):e0251128. doi: 10.1371/journal.pone.0251128 (PMC8112677; doi:10.1371/journal.pone.0251128)
Supplement: S1 File — (PDF) [file pone.0251128.s001.pdf]

Current SLT Users Interview Guide

1. **INTRODUCTION**
   1.1 The purpose of this meeting is to discuss thoughts and opinions related to tobacco use among firefighters.
   1.2. Your comments will remain confidential. We have asked for your demographic information for descriptive purposes only. We will not report any individual comments tied to you in our research reports. We are digitally recording the conversation so that we can transcribe what you say and see if there are similar themes about this topic across focus groups/interviews. We will not identify you in any transcribed notes from this group. If you have any questions about how the information you provide will be used, you can ask now or contact Dr. Jitnarin.
   1.3. Process
      1.3.1. Express your own thoughts and opinions
      1.3.2. We want to hear positive and negative comments
      1.3.3. There are no wrong answers; we expect different opinions
2. **PERCEPTIONS OF CANCER RISK**
   2.1. What do you think your chance is of developing cancer while you are in the fire service?
   2.2. How would you rate your chance of developing cancer, and how do you think your chance of developing cancer compares to the average people your age?
   2.3. What has contributed to the cancer risk factors among firefighters?
   2.4. Do you think there is an association between cancer risk and SLT use?
3. **TOBACCO USE IN THE FIRE SERVICE**
   3.1. How common/acceptable is tobacco in the fire service?
   3.2. How common/acceptable is smokeless tobacco use in particular in the fire service?
4. **SLT HISTORY**
   4.1. How old were you when you started using SLT?
   4.2. How often and how much do you use?
5. **SLT INITIATION**
   5.1. When did you start using SLT regularly? (probes: before/after joined the fire service)
   5.2. What factors contributed to your choosing to SLT? (probes: peers, family, hobbies)
6. **FIRE SERVICE & SLT**
   6.1. How common is SLT use among the personnel at your department?
   6.2. What factors about the fire service/your department encourage SLT use?
   6.3. What factors about the fire service/your department encourage SLT use?
7. **SLT CESSATION**
   7.1. Have you considered quitting SLT?
   If yes….
      7.2. What has made you want to quit?
      7.3. What methods did you use to help you stop using SLT?
      7.4. What cessation methods were useful/not useful?
      7.5. What is most difficult about quitting?
   If no…
      7.6. Do you think you will be using 5 years from now? The rest of your life?
      7.7. What do you enjoy about using SLT?
      7.8. What do you not like about SLT?
      7.9. What reasons would make you want to quit?
   7.2 How could firefighters benefit from technology based (e.g., web, mobile, texting, email) interventions?
   7.3 What are the technology-based strategies might help you quit SLT?

8. DESIGNING CESSATION AIDS

8.1 If you were tasked with developing a SLT cessation program for the fire service, what would you include? (Probes: format, incentives, educational information, technology – text messaging, email, apps)

Former SLT Users Interview Guide

1. **INTRODUCTION**
   1.1 The purpose of this meeting is to discuss thoughts and opinions related to tobacco use among firefighters.
   1.2 Your comments will remain confidential. We have asked for your demographic information for descriptive purposes only. We will not report any individual comments tied to you in our research reports. We are digitally recording the conversation so that we can transcribe what you say and see if there are similar themes about this topic across focus groups/interviews. We will not identify you in any transcribed notes from this group. If you have any questions about how the information you provide will be used, you can ask now or contact Dr. Jitnarin.
   1.3 Process
      1.3.1. Express your own thoughts and opinions
      1.3.2 We want to hear positive and negative comments
      1.3.3 There are no wrong answers; we expect different opinions
2. **PERCEPTIONS OF CANCER RISK**
   2.1. What do you think your chance is of developing cancer while you are in the fire service?
   2.2. How would you rate your chance of developing cancer, and how do you think your chance of developing cancer compares to the average people your age?
   2.3. What has contributed to the cancer risk factors among firefighters?
   2.4. Do you think there is an association between cancer risk and SLT use?
3. **TOBACCO USE IN THE FIRE SERVICE**
   3.1. How common/acceptable is tobacco in the fire service?
   3.2. How common/acceptable is smokeless tobacco use in particular in the fire service?
4. **SLT HISTORY**
   4.1. How old were you when you started using SLT?
   4.2. How long did you use SLT?
5. **SLT INITIATION**
   5.1. When did you start using SLT regularly? (probes: before/after joined the fire service)
   5.2. What factors contributed to your choosing to SLT? (probes: peers, family, hobbies)
6. **FIRE SERVICE & SLT**
   6.1. How common is SLT use among the personnel at your department?
   6.2. What factors about the fire service/your department encourage SLT use?
   6.3. What factors about the fire service/your department encourage SLT use?
7. **SLT CESSATION**
   7.1. What reasons led to the decision to stop using SLT?
   7.2. What methods did you use to help you stop using SLT?
   7.3. What cessation methods were useful?
   7.4. What cessation methods were not useful?
   7.5 How would a technology based SLT intervention be perceived by firefighters??
   7.6 What are the technology-based strategies that you think firefighters might want for quitting SLT?
8. **DESIGNING CESSATION AIDS**

8.1 If you were tasked with developing a SLT cessation program for the fire service, what would you include? (Probes: format, incentives, educational information, technology – text messaging, email, apps).

INFORMED CONSENT
Cancer Risk and the Epidemic of Smokeless Tobacco in the US Fire Service

Investigative Team
Principal Investigator: Nattinee Jitnarin, Ph.D.
Co-Investigators: C. Keith Haddock, Ph.D.
Walker S.C. Poston, Ph.D.
Sara Jahnke, Ph.D.

Institute for Biobehavioral Health Research
National Development & Research Institute (NDRI)

Invitation to Participate and Purpose
You are invited to participate in a research study of the opinions and attitudes of fire service leaders and firefighters about the perceptions on occupational risks for cancer and smokeless tobacco (SLT) cessation as well as the most effective messages and methodologies for intervening with firefighters. You are being encouraged to participate because of your role in fire service. About 30 individuals will be asked to participate in this study. Please take your time to read this document and make your decision about participating. If you have any questions, you may ask the researchers.

Description of Procedures
The interview will take approximately 30 to 60 minutes. Digital files will be used to record the discussion; however, neither your name nor any uniquely identifying information will be intentionally recorded. Following the interview, written transcripts will be made of the audio tapes. We will attempt to remove any uniquely identifying information in the transcripts in order to protect your confidentiality. Digital files will be erased 3 years from the date of the interview and only transcribed files without identifying information will be retained. You also will be asked to complete a demographic questionnaire as part of the study. However, your answers to the demographic questionnaire will not be linked to your responses during the interview.

Voluntary Participation
Participation in this study is voluntary at all times. You may choose to not participate or to withdraw your participation at any time. Deciding not to participate or choosing to leave the study will not result in any penalty. You may refuse to answer any questions. If you withdraw from the study, information you have already provided will be used for the study and remain confidential.

Fees and Expenses
There will be no expenses incurred on your part as a result of participating in this study.

Risks/Inconveniences and Benefits
There are no known risks associated with this study. We cannot and do not guarantee or promise that you will receive any benefits from this study. Your decision whether or not to participate in this study will not affect your employment.

Confidentiality
While every effort will be made to keep confidential all of your information confidential it cannot be absolutely guaranteed. Individuals from NDRI Institutional Review Board (a committee that reviews and approves research studies), Research Protections Program, and Federal regulatory agencies may look at records related to this study for quality improvement and regulatory functions. Only the principal investigator and immediate study personnel (i.e. co-investigators, research assistants) will have access to your information. Unique identifiers will be maintained in a separate file which will be available only to project personnel. All information will be stored in a secured facility.

Questions
If you have any questions concerning this study, or to report any possible injury that might occur as a result of your participation in this study, please feel free to contact Dr. Jitnarin at 913-681-0300. You may contact Lisa Bernhard at (888) 845-4695 to answer any questions you may have about your rights as a research subject. No provision has been made to pay any subject for harm which may result from participation, but nothing in this consent limits your right to seek payment for any harm resulting from your participation.

Verbal Consent for Participation in a Research Study

PARTICIPATION IN RESEARCH IS VOLUNTARY. You have the right to decline to be in this study, or to withdraw from it at any point without penalty or loss of benefits to which you are otherwise entitled.

Before beginning the interview, please let the study scientists know whether you have read this document. If you have any questions please ask them before beginning the interview.

If you have read this document and have no questions, please let the project scientists know if you agree to participate in this study.
