# Supplementary material for: A qualitative study to assess perceptions, barriers, and motivators supporting smokeless tobacco cessation in the US fire service
Source: PLoS One. 2021 May 11;16(5):e0251128. doi: 10.1371/journal.pone.0251128 (PMC8112677; doi:10.1371/journal.pone.0251128)
Supplement: S2 File — (DOCX) [file pone.0251128.s002.docx]

**Manuscript:** A qualitative study to assess perceptions, barriers, and motivators supporting smokeless tobacco cessation in the US fire service.

**Consolidated criteria for reporting qualitative studies (COREQ): 32-item checklist**

Developed from: Tong A, Sainsbury P, Craig J. Consolidated criteria for reporting qualitative research (COREQ): a 32-item checklist for interviews and focus groups. *International Journal for Quality in Health Care*. 2007. Volume 19, Number 6: pp. 349 – 357.

| **No. Item** | **Guide questions/description** | **Reported on Page #** |
| --- | --- | --- |
| **Domain 1: Research team and reﬂexivity** |  |  |
| *Personal Characteristics* |  |  |
| 1. Interviewer/facilitator | Which author/s conducted the interview or focus group? | NJ, CP, HK (p. 7) |
| 2. Credentials | What were the researcher’s credentials? E.g. PhD, MD | PhD/BSc (title page) |
| 3. Occupation | What was their occupation at the time of the study? | Researchers |
| 4. Gender | Was the researcher male or female? | Females/Males |
| 5. Experience and training | What experience or training did the researcher have? | Official/Field training |
| *Relationship with participants* |  |  |
| 6. Relationship established | Was a relationship established prior to study commencement? | Relationship was present with some of the participants, but otherwise relationship was not established prior to interviews. |
| 7. Participant knowledge of the interviewer | What did the participants know about the researcher? e.g. personal goals, reasons for doing the research | Participants were informed about the reason of this research (p. 6). |
| 8. Interviewer characteristics | What characteristics were reported about the interviewer/facilitator? e.g. Bias, assumptions, reasons and interests in the research topic | Description of interviewers’ experience and work were provided prior to the interviews. Participants knew that we were interested in learning about participants’ opinion and perceptions toward SLT use and cessation (p. 6-7) |
| **Domain 2: study design** |  |  |
| *Theoretical framework* |  |  |
| 9. Methodological orientation and Theory | What methodological orientation was stated to underpin the study? e.g. grounded theory, discourse analysis, ethnography, phenomenology, content analysis | A social cognitive theory (SCT) domain analysis and a grounded theory approach (p. 7-8) |
| *Participant selection* |  |  |
| 10. Sampling | How were participants selected? e.g. purposive, convenience, consecutive, snowball | Purposeful random (p. 6) |
| 11. Method of approach | How were participants approached? e.g. face-to-face, telephone, mail, email | Email, telephone (p. 6-7) |
| 12. Sample size | How many participants were in the study? | 23 (p. 6) |
| 13. Non-participation | How many people refused to participate or dropped out? Reasons? | The study did not have any participants subsequently drop out. There were also no potential participants who refused to participante after being contacted. |
| *Setting* |  |  |
| 14. Setting of data collection | Where was the data collected? e.g. home, clinic, workplace | Individual phone interviews took place selected by participants (p. 6-7) |
| 15. Presence of non-participants | Was anyone else present besides the participants and researchers? | No (p. 7) |
| 16. Description of sample | What are the important characteristics of the sample? e.g. demographic data, date | Male and mostly White as reported in Table 1 (p. 9). |
| *Data collection* |  |  |
| 17. Interview guide | Were questions, prompts, guides provided by the authors? Was it pilot tested? | Interview guide (reported in S1 File) |
| 18. Repeat interviews | Were repeat inter views carried out? If yes, how many? | No |
| 19. Audio/visual recording | Did the research use audio or visual recording to collect the data? | All interviews were audio-recorded with participant consent (p. 7) |
| 20. Field notes | Were ﬁeld notes made during and/or after the interview or focus group? | Yes (p. 7) |
| 21. Duration | What was the duration of the inter views or focus group? | Ranging from 20-50 minutes with median of 42 minutes (p. 7) |
| 22. Data saturation | Was data saturation discussed? | Yes (p. 6-7) |
| 23. Transcripts returned | Were transcripts returned to participants for comment and/or correction? | No |
| **Domain 3: analysis and ﬁndings** |  |  |
| *Data analysis* |  |  |
| 24. Number of data coders | How many data coders coded the data? | Two (p. 8) |
| 25. Description of the coding tree | Did authors provide a description of the coding tree? | No. We discussed on p. 7-8 in the methods that we used a SCT domain analysis and a grounded theory approach to code and analyzed the data. |
| 26. Derivation of themes | Were themes identiﬁed in advance or derived from the data? | Themes were derived from the data (p. 8). |
| 27. Software | What software, if applicable, was used to manage the data? | NVivo 12 (p. 8) |
| 28. Participant checking | Did participants provide feedback on the ﬁndings? | No |
| *Reporting* |  |  |
| 29. Quotations presented | Were participant quotations presented to illustrate the themes/ﬁndings? Was each quotation identiﬁed? e.g. participant number | Yes (p. 9-21) |
| 30. Data and ﬁndings consistent | Was there consistency between the data presented and the ﬁndings? | Yes (p. 9-21) |
| 31. Clarity of major themes | Were major themes clearly presented in the ﬁndings? | Yes (p. 9-21) |
| 32. Clarity of minor themes | Is there a description of diverse cases or discussion of minor themes? | Yes (p. 9-21) |
