# Supplementary material for: A qualitative study to assess perceptions, barriers, and motivators supporting smokeless tobacco cessation in the US fire service
Source: PLoS One. 2021 May 11;16(5):e0251128. doi: 10.1371/journal.pone.0251128 (PMC8112677; doi:10.1371/journal.pone.0251128)
Supplement: Attachment — Submitted filename: Review_PONE-D-20-18503_15July2020.docx [file pone.0251128.s003.docx]

**Review: “A qualitative study to assess perceptions, barriers, and motivators supporting smokeless tobacco cessation in the US fire service”**

This paper reports on findings from a qualitative study that explored firefighters’ experiences and perceptions of smokeless tobacco (SLT) use, as well as their motivations and barriers to SLT cessation. A strength of the study is that it is the first qualitative study to specifically explore firefighters’ experiences with SLT use and cessation, and firefighters are a group with particularly high rates of SLT use. I appreciate how much time goes into qualitative research and commend the authors for their hard work. There are, however, several major drawbacks in the current version of this paper, which I have outlined below with suggestions for revision.

**(1) The worthiness of the topic is not well established.**

The current introduction does not contextualize the research problem within the relevant existing literature, so the reader cannot be convinced that this is a problem worthy of study or imagine the practical or theoretical impact of the reported findings. The reader is left with many questions:

What are the negative effects of smokeless tobacco use among fire fighters? In other words, why should we care if rates of use are high within this population? The introduction currently reviews the health effects of combustible tobacco.

Have non-tailored SLT cessation interventions been implemented among firefighters in the past? If so, how successful were they? If none were implemented, what reasons can the authors provide to support their statement that tailored SLT interventions are necessary for this group rather than non-tailored interventions that already exist? In other words, if the justification for this study is that it can inform the development of tailored interventions for this group, a stronger case for the need for tailored interventions needs to be made in the introduction.

Relatedly, why should we think that fire fighters would have unique perceptions/experiences regarding SLT as compared to other groups like construction workers or emergency medical technicians/paramedics? Why would we think that fire fighters’ perceptions/experiences with SLT would be different from their perceptions/experiences concerning combustible tobacco?

I think answering these questions in the introduction will help to narrow and define the research problem and establish it as a more worthy and significant topic.

**(2) As it is currently presented, there is a lack of methodological coherence in how the study was designed, how the interview guide was developed, and the analytic strategy in deriving themes from the data.**

The sampling strategy is not well justified. For example, why include both current and former users in the same analysis? Wouldn’t a former user who has successfully quit potentially have different motivators and barriers to quitting than someone who has been using SLT for many years, tried to quit many times, and not been successful in doing so?

Why was the interview guide developed with Social Cognitive Theory? The first mention of SCT comes a bit out of the blue in the methods section. No explanation of what SCT is or justification for its application to the interview guide is given. There is also mention of ‘previous literature’ that informed how the interview guide was developed. What findings/conceptual frameworks were taken from previous literature to help develop the interview guide?

The paper states that interviews ranged from 20 to 50 minutes. It does not seem credible that a meaningful discussion concerning all of the domains listed in the interview guide could be had in 20 minutes. This casts doubt on the quality of the data.

As a reader, I experienced confusion regarding the analytic plan and how themes were derived from the data. For example, it appears that the transcripts were already partially coded and a coding scheme was developed prior to the application of a ‘SCT domain analysis and grounded theory’ approach. My understanding is that a methodological orientation, like grounded theory, should guide the methodological approach from the very beginning, rather than be introduced in the middle of the analytic process.

The paper states that “a thematic analysis by searching for patterns and themes that frequently occurred in a single interview or were common across interviews” was conducted. These sound like different analytic strategies. What was the rationale behind this?

If Social Cognitive Theory and ‘previous literature’ were used to develop the interview guide, I would expect these to also appear in the analytic strategy. Were some themes identified in advance to guide coding the transcripts (and if so these should be made explicit), or were themes derived entirely from the data?

**(3) The findings regarding participants’ experiences with and perceptions of SLT product use did not strike this reader as particularly novel.**

These included participants’ perceptions of reduced health risk with SLT use, the discretion and convenience of SLT products, and the use of SLT to cope with boredom and stress on the job. See, for example, young adults’ reported perceptions and experiences with smokeless tobacco in: McQuoid J, Keamy-Minor E, Ling PM. A practice theory approach to understanding poly-tobacco use. Critical Public Health. 2018 Nov 01. 10.1080/09581596.2018.1541226.

The findings regarding the specific motivators for and barriers to quitting SLT for firefighters struck me as more of a contribution to the literature and seemed relevant to the development of a tailored SLT cessation intervention for fire fighters. I would emphasize these findings more in the next version of the paper. In particular:

- Some firefighters worried that withdrawal would distract them from performing firefighting tasks
- The use of SLT to develop/strengthen camaraderie between firefighters
- Lack of support to quit SLT from health and other service providers. Concerns about the limited SLT specific cessation treatments, as well as a lack of restrictions on SLT use in the departments.
- Unique stressors of fire fighting
- Receptivity to an incentives program for quitting
- Sensitivity to SLT price increases

**(4) There is sometimes a lack of clarity and accuracy in how terms and constructs are used in the paper.**

In one example, the abstract refers to “the belief that it was effective for coping with boredom and stress”. This appears to be a statement of something that fire fighters have empirically experienced, so the term ‘belief’ does not seem appropriate.
