# Supplementary material for: A qualitative study to assess perceptions, barriers, and motivators supporting smokeless tobacco cessation in the US fire service
Source: PLoS One. 2021 May 11;16(5):e0251128. doi: 10.1371/journal.pone.0251128 (PMC8112677; doi:10.1371/journal.pone.0251128)
Supplement: Attachment — Submitted filename: Responses to Reviewers.docx [file pone.0251128.s004.docx]

**Our responses to the editor and reviewers are in bold texts below:**

*Thank you for submitting your manuscript to PLOS ONE. After careful consideration, we feel that it has merit but does not fully meet PLOS ONE’s publication criteria as it currently stands. Therefore, we invite you to submit a revised version of the manuscript that addresses the points raised during the review process.*

*In revising the paper, please pay special attention to the methodological issues raised by Reviewer 2's comments [2] and [4]. Per PLOS ONE's editorial policies, novelty is not an issue in the decision of whether or not to accept the paper, although the paper would be strengthened by making what is new in the paper clearer. You should also address Reviewer 1's suggestions.*
 **We would like to thank the Academic Editor for the careful reading, and the reviewers for their high quality and constructive review of our manuscript. We have revised and edited the manuscript in response to the extensive and insightful reviewers’ comments. We hope that a revised version of the manuscript will be considered by PLOS ONE.**

*Please submit your revised manuscript by Sep 11 2020 11:59PM. If you will need more time than this to complete your revisions, please reply to this message or contact the journal office at* [*plosone@plos.org*](mailto:plosone@plos.org)*. When you're ready to submit your revision, log on to* [*https://www.editorialmanager.com/pone/*](https://www.editorialmanager.com/pone/) *and select the 'Submissions Needing Revision' folder to locate your manuscript file.

Please include the following items when submitting your revised manuscript:*

- *A rebuttal letter that responds to each point raised by the academic editor and reviewer(s). You should upload this letter as a separate file labeled 'Response to Reviewers'.*
- *A marked-up copy of your manuscript that highlights changes made to the original version. You should upload this as a separate file labeled 'Revised Manuscript with Track Changes'.*
- *An unmarked version of your revised paper without tracked changes. You should upload this as a separate file labeled 'Manuscript'.*

**We now include this rebuttal letter, the marked-up copy of our manuscript with track changes indicating edits made per the reviewers’ suggestions, and the unmarked version of our revised manuscript.**

*If you would like to make changes to your financial disclosure, please include your updated statement in your cover letter. Guidelines for resubmitting your figure files are available below the reviewer comments at the end of this letter.

If applicable, we recommend that you deposit your laboratory protocols in* [*protocols.io*](http://protocols.io/) *to enhance the reproducibility of your results. Protocols.io assigns your protocol its own identifier (DOI) so that it can be cited independently in the future. For instructions see:* [*http://journals.plos.org/plosone/s/submission-guidelines#loc-laboratory-protocols*](http://journals.plos.org/plosone/s/submission-guidelines#loc-laboratory-protocols) *Journal Requirements:*

*When submitting your revision, we need you to address these additional requirements.*

*1. Please ensure that your manuscript meets PLOS ONE's style requirements, including those for file naming. The PLOS ONE style templates can be found at*

[*https://journals.plos.org/plosone/s/file?id=wjVg/PLOSOne_formatting_sample_main_body.pdf*](https://journals.plos.org/plosone/s/file?id=wjVg/PLOSOne_formatting_sample_main_body.pdf) 
*and*
[*https://journals.plos.org/plosone/s/file?id=ba62/PLOSOne_formatting_sample_title_authors_affiliations.pdf*](https://journals.plos.org/plosone/s/file?id=ba62/PLOSOne_formatting_sample_title_authors_affiliations.pdf)

**We have revised and reformatted our manuscript and its supporting information files to meet PLOS ONE’s style requirements.**

*2. Please note that according to our submission guidelines (*[*http://journals.plos.org/plosone/s/submission-guidelines*](http://journals.plos.org/plosone/s/submission-guidelines)*), outmoded terms and potentially stigmatizing labels should be changed to more current, acceptable terminology. For example: “Caucasian” should be changed to “white” or “of [Western] European descent” (as appropriate).*

**We changed “Caucasian” to “White” in Table 1.**

*3. In your Data Availability statement, you have not specified where the minimal data set underlying the results described in your manuscript can be found. PLOS defines a study's minimal data set as the underlying data used to reach the conclusions drawn in the manuscript and any additional data required to replicate the reported study findings in their entirety. All PLOS journals require that the minimal data set be made fully available. For more information about our data policy, please see* [*http://journals.plos.org/plosone/s/data-availability*](http://journals.plos.org/plosone/s/data-availability)*.*

*Upon re-submitting your revised manuscript, please upload your study’s minimal underlying data set as either Supporting Information files or to a stable, public repository and include the relevant URLs, DOIs, or accession numbers within your revised cover letter. For a list of acceptable repositories, please see* [*http://journals.plos.org/plosone/s/data-availability#loc-recommended-repositories*](http://journals.plos.org/plosone/s/data-availability#loc-recommended-repositories)*. Any potentially identifying patient information must be fully anonymized.*

*Important: If there are ethical or legal restrictions to sharing your data publicly, please explain these restrictions in detail. Please see our guidelines for more information on what we consider unacceptable restrictions to publicly sharing data:* [*http://journals.plos.org/plosone/s/data-availability#loc-unacceptable-data-access-restrictions*](http://journals.plos.org/plosone/s/data-availability#loc-unacceptable-data-access-restrictions)*. Note that it is not acceptable for the authors to be the sole named individuals responsible for ensuring data access.*

*We will update your Data Availability statement to reflect the information you provide in your cover letter.*

**We clarified and updated our Data Availability statement in our cover letter. Our data cannot be shared publicly because participant consent was not given for interview transcripts to be shared. However, requests for anonymized data can be made to the Assistant Grants Management Director Carol Tarzian (contact via tarzian@ndri-usa.org).**

*4. Please include captions for your Supporting Information files at the end of your manuscript, and update any in-text citations to match accordingly. Please see our Supporting Information guidelines for more information: http://journals.plos.org/plosone/s/supporting-information.*

**We now include captions for our Supporting Information files at the end of our manuscript and have updated in-text citations accordingly.**

**S1 File. Former and current SLT users interview guide with consent form.
S2 File. Consolidated criteria for reporting qualitative research (COREQ) checklist.**

*[Note: HTML markup is below. Please do not edit.]

Reviewers' comments:

Reviewer's Responses to Questions*

***Comments to the Author*** *1. Is the manuscript technically sound, and do the data support the conclusions?

The manuscript must describe a technically sound piece of scientific research with data that supports the conclusions. Experiments must have been conducted rigorously, with appropriate controls, replication, and sample sizes. The conclusions must be drawn appropriately based on the data presented.*

*Reviewer #1: No*

*Reviewer #2: Yes*

*2. Has the statistical analysis been performed appropriately and rigorously?*

*Reviewer #1: N/A*

*Reviewer #2: Yes*

*3. Have the authors made all data underlying the findings in their manuscript fully available?

The* [*PLOS Data policy*](http://www.plosone.org/static/policies.action#sharing) *requires authors to make all data underlying the findings described in their manuscript fully available without restriction, with rare exception (please refer to the Data Availability Statement in the manuscript PDF file). The data should be provided as part of the manuscript or its supporting information, or deposited to a public repository. For example, in addition to summary statistics, the data points behind means, medians and variance measures should be available. If there are restrictions on publicly sharing data—e.g. participant privacy or use of data from a third party—those must be specified.*

*Reviewer #1: No*

*Reviewer #2: Yes*

*4. Is the manuscript presented in an intelligible fashion and written in standard English?

PLOS ONE does not copyedit accepted manuscripts, so the language in submitted articles must be clear, correct, and unambiguous. Any typographical or grammatical errors should be corrected at revision, so please note any specific errors here.*

*Reviewer #1: Yes*

*Reviewer #2: Yes*

*5. Review Comments to the Author

Please use the space provided to explain your answers to the questions above. You may also include additional comments for the author, including concerns about dual publication, research ethics, or publication ethics. (Please upload your review as an attachment if it exceeds 20,000 characters)*

**We would like to thank the reviewers for their high quality and constructive reviews of our manuscript. We have revised and modified our manuscript per the reviewers’ comments and suggestions. Detailed point-by-point responses to each of the reviewers’ comments are follow:**

*Reviewer #1: This paper reports on findings from a qualitative study that explored firefighters’ experiences and perceptions of smokeless tobacco (SLT) use, as well as their motivations and barriers to SLT cessation. A strength of the study is that it is the first qualitative study to specifically explore firefighters’ experiences with SLT use and cessation, and firefighters are a group with particularly high rates of SLT use. I appreciate how much time goes into qualitative research and commend the authors for their hard work. There are, however, several major drawbacks in the current version of this paper, which I have outlined with suggestions for revision in the attached comments.*

**We copied the reviewer#1 suggestions from the attached file and responded to their comments below. We thank the reviewer for their suggestions and now substantially edited and adjusted our manuscript per their comments.**

*This paper reports on findings from a qualitative study that explored firefighters’ experiences and perceptions of smokeless tobacco (SLT) use, as well as their motivations and barriers to SLT cessation. A strength of the study is that it is the first qualitative study to specifically explore firefighters’ experiences with SLT use and cessation, and firefighters are a group with particularly high rates of SLT use. I appreciate how much time goes into qualitative research and commend the authors for their hard work. There are, however, several major drawbacks in the current version of this paper, which I have outlined below with suggestions for revision.*

*(1) The worthiness of the topic is not well established.*

*The current introduction does not contextualize the research problem within the relevant existing literature, so the reader cannot be convinced that this is a problem worthy of study or imagine the practical or theoretical impact of the reported findings. The reader is left with many questions:*

*What are the negative effects of smokeless tobacco use among fire fighters? In other words, why should we care if rates of use are high within this population? The introduction currently reviews the health effects of combustible tobacco.*

**We now provided additional details on the negative effects of SLT on firefighters.**

*Have non-tailored SLT cessation interventions been implemented among firefighters in the past? If so, how successful were they? If none were implemented, what reasons can the authors provide to support their statement that tailored SLT interventions are necessary for this group rather than non-tailored interventions that already exist? In other words, if the justification for this study is that it can inform the development of tailored interventions for this group, a stronger case for the need for tailored interventions needs to be made in the introduction.*

**We provided additional explanations to support the statements that tailored SLT cessation programs for firefighters are necessary and needed to be implemented.**

*Relatedly, why should we think that fire fighters would have unique perceptions/experiences regarding SLT as compared to other groups like construction workers or emergency medical technicians/paramedics? Why would we think that fire fighters’ perceptions/experiences with SLT would be different from their perceptions/experiences concerning combustible tobacco?*

**A number of reasons were considered for the unique cultures in fire service that may influence firefighters’ opinions and perspectives on SLT use including their long work hours (24-hour shifts or longer), the social and work bonding that occurs around their on-duty and off-duty days, and the stress associated with firefighting tasks. Although other occupations the reviewer mentioned have shift working structure similar to firefighters, they do not spend time (both live and work) at the same place in long hours like firefighters do in their fire station. Further, the significantly higher rate of SLT use compared to these other occupational groups and the general population rates suggest something specific to the fire service that is promoting use.**

**Results from our previous study on tobacco use in a fire service personnel (Poston et al., 2012) showed that firefighters viewed and/or perceived SLT use and cigarette use (combustible tobacco) differently. For example, many firefighters noted that smoking negatively impacts their health and fitness and less acceptable among peer compared to SLT. Our findings from the current study also supported that SLT has been viewed positively and is viewed as acceptable in fire house more than cigarette smoking.**

*I think answering these questions in the introduction will help to narrow and define the research problem and establish it as a more worthy and significant topic.*

**We revised the introduction section to address the reviewer’s concerns.**

*(2) As it is currently presented, there is a lack of methodological coherence in how the study was designed, how the interview guide was developed, and the analytic strategy in deriving themes from the data.*

*The sampling strategy is not well justified. For example, why include both current and former users in the same analysis? Wouldn’t a former user who has successfully quit potentially have different motivators and barriers to quitting than someone who has been using SLT for many years, tried to quit many times, and not been successful in doing so?*

**Data were analyzed separately for each group (current vs. former SLT users). We found similar themes across groups thus we presented results derived from both groups under the same themes. We added a sentence to clarify that the transcripts from current and former SLT users were coded and analyzed separately.**

*Why was the interview guide developed with Social Cognitive Theory? The first mention of SCT comes a bit out of the blue in the methods section. No explanation of what SCT is or justification for its application to the interview guide is given. There is also mention of ‘previous literature’ that informed how the interview guide was developed. What findings/conceptual frameworks were taken from previous literature to help develop the interview guide?*

**We have added the rationale/justification of using SCT in the introduction section and provided more details on using SCT for developing the interview guides in the method section. Findings from previous literature that were used to help develop the interview guide also are provided.**

*The paper states that interviews ranged from 20 to 50 minutes. It does not seem credible that a meaningful discussion concerning all of the domains listed in the interview guide could be had in 20 minutes. This casts doubt on the quality of the data.*

**In one interview, we had to stop the interview early (20 minutes interview) due to participant’s responding to a fire call. To avoid the misunderstanding and resulting confusion, we now only report the average length of the interview.**

*As a reader, I experienced confusion regarding the analytic plan and how themes were derived from the data. For example, it appears that the transcripts were already partially coded and a coding scheme was developed prior to the application of a ‘SCT domain analysis and grounded theory’ approach. My understanding is that a methodological orientation, like grounded theory, should guide the methodological approach from the very beginning, rather than be introduced in the middle of the analytic process.*

**We revised the analytic plan and now added sentences to introduce the methodological approach at the beginning of data analysis section.**

*The paper states that “a thematic analysis by searching for patterns and themes that frequently occurred in a single interview or were common across interviews” was conducted. These sound like different analytic strategies. What was the rationale behind this?*

**In this step, we aimed to explore themes or patterns that frequently noted or occurred across interviews or groups. For example, under the existing SLT cessation services/supports in the fire service domain, both current and former SLT users frequently mentioned lack of support from their healthcare providers and from their fire departments regarding SLT cessation programs.**

*If Social Cognitive Theory and ‘previous literature’ were used to develop the interview guide, I would expect these to also appear in the analytic strategy. Were some themes identified in advance to guide coding the transcripts (and if so these should be made explicit), or were themes derived entirely from the data?*

**We added an explanation on the use of SCT and how previous literature was used to develop the interview guide. We also added context about the domains that identified in advance to guide coding the transcripts.**

*(3) The findings regarding participants’ experiences with and perceptions of SLT product use did not strike this reader as particularly novel.*

*These included participants’ perceptions of reduced health risk with SLT use, the discretion and convenience of SLT products, and the use of SLT to cope with boredom and stress on the job. See, for example, young adults’ reported perceptions and experiences with smokeless tobacco in: McQuoid J, Keamy-Minor E, Ling PM. A practice theory approach to understanding poly-tobacco use. Critical Public Health. 2018 Nov 01. 10.1080/09581596.2018.1541226.*

**Although some of the findings were similar to those found in the general population, we believe that the current study addresses a key gap in the occupational health literature with a group whose SLT rates are substantially higher than the US general population. We also believe the findings from this research will form the basis of developing an effective intervention to assist firefighters quit using SLT.**

*The findings regarding the specific motivators for and barriers to quitting SLT for firefighters struck me as more of a contribution to the literature and seemed relevant to the development of a tailored SLT cessation intervention for fire fighters. I would emphasize these findings more in the next version of the paper. In particular:*

- *Some firefighters worried that withdrawal would distract them from performing firefighting tasks*
- *The use of SLT to develop/strengthen camaraderie between firefighters*
- *Lack of support to quit SLT from health and other service providers. Concerns about the limited SLT specific cessation treatments, as well as a lack of restrictions on SLT use in the departments.*
- *Unique stressors of fire fighting*
- *Receptivity to an incentives program for quitting*
- *Sensitivity to SLT price increases*

**We revised our manuscript and emphasized the findings the reviewer’s raised in both results and discussion sections.**

***(4) There is sometimes a lack of clarity and accuracy in how terms and constructs are used in the paper.***

*In one example, the abstract refers to “the belief that it was effective for coping with boredom and stress”. This appears to be a statement of something that fire fighters have empirically experienced, so the term ‘belief’ does not seem appropriate.*

**We have changed the term “belief” and revised the sentence in the abstract. We also edited the manuscript in line with the reviewer’s comments.**

*Reviewer #2: This is well designed and well-presented qualitative study on the use of smokeless tobacco among career firefighters in the US. It is an important topic as there is now strong evidence that firefighters in the US and elsewhere are at increased risk for some cancer sites, and there is an emerging body of literature on elucidating possible sources of that risk as well as prevention and control strategies that address firefighting culture. There are minor weaknesses to be addressed.*

**We thank the reviewer for their suggestion and now address the weaknesses raised by the reviewer.**
 *Introduction

• Starting on line 67: The Union is generally focused on Reducing Tobacco Use - not just smoking cessation, please revise the text to reflect this.*

**This is revised in the current version.**  *• The background on US workers rate of SLT use is missing some relevant information, specifically, (starting on line 73) “For example, we found that between 10.5%-18.4% of male firefighters exclusively used SLT which is almost three times the rate of SLT use found in the general population [13] and higher than any occupational group reported for the U.S. civilian workforce [17].” Citation 17 is Dietz et al., 2011 who present findings on four aggregated sectors of the US workers. However, three more recent studies that present findings from analysis of BRFSS, NSDUH, and NHIS data, each explore more refined worker sectors. All three of these studies reported that SLT use among extraction workers was similar, or higher, then the 10.5%-18.4% rate SLT among firefighters (please see citations below). This has important implications in terms of possible relevance of this paper to the extraction sector, particularly the recommendation for SLT cessation-treatment be tailored to the particular culture of firefighters (the authors do an excellent job of addressing this point) could be extended as a more general statement for other workforces.

Citations about SLT prevalence the extraction industry:
• Kristin Yeoman K, Sussell A, Retzer K , Poplin G. Health Risk Factors Among Miners, Oil and Gas Extraction Workers, Other Manual Labor Workers, and Nonmanual Labor Workers, BRFSS 2013-2017, 32 States. Workplace Health Saf. 2020 Aug;68(8):391-401. doi: 10.1177/2165079920909136. Epub 2020 Jun 2.
• Graber JM, Delnevo CD, Manderski MT, et al. Cigarettes, Smokeless Tobacco, and Poly-Tobacco Among Workers in Three Dusty Industries. J Occup Environ Med. 2016 May;58(5):477-84. doi: 10.1097/JOM.0000000000000699. PMID: 27158955
• Mazurek JM, Syamlal G, King BA, Castellan RM, Smokeless tobacco use among working adults - United States, 2005 and 2010. MMWR Morb Mortal Wkly Rep. 2014 Jun 6;63(22):477-82. PMID: 24898164 PMCID: PMC5779362*

**We revised the sentences and updated the citations to reflect the current findings on SLT prevalence in other occupational groups.**  *Methods
• Overall a very nice presentation and an unusually good balance with the amount and choice of quotes to support/demonstrate the themes. A few suggested changes:
o the 3 quotes for the ‘Social and community networks’ subheading (page 20, starting line 272) – this seems like a lot and the first quote seems to describe stress relief better than connectivity.
o on page 21, line 293 The quote could be shortened without impacting the content by deleting “The EAP does not have…a tobacco cessation program.” And replacing with “…”
o For the quote string on Page 23, line 336, consider defining “it” parenthetically with something like “[using SLT]”
• Was dual use of SLT and combustible tobacco explored? While smoking is bit lower among firefighters than the general public (cited here 13.2% vs 15.6%), dual tobacco use in an occupational setting is an important topic since smoking is much more effective nicotine-delivery methods and people who chew at work may smoke off shift. This may not be given as a reason for chewing at work by career firefighters without prompting given that there has been cultural shift away from combustible tobacco use*

**We have edited the Methods section per the reviewer’s suggestions. We deleted the first quote under the “Social and community networks” subheading to make the section shorter. We also replaced *“The EAP does not have…a tobacco cessation program.”* with *“…”*, and added [using SLT] to refer to “it”.**

*• The prevalence of SLT use varies significantly by region in the US, with highest rates in the South and West. This was national study with representation in 3 regions - (East, Central, and West). Were the authors able to see if there were differences on the cultural acceptance of SLT use within the fire service by region?*

**Although SLT use in the general population varies significantly by region, our previous qualitative work in the fire service suggests that, although there are some regional differences for certain issues, there is a core common culture in the Fire Service, particularly in regards to substance use, particularly SLT [Poston WSC, Haddock CK, Jitnarin N, Jahnke SA. A national qualitative study of tobacco use among career firefighters. Nicotine Tob Res 2012;14:734-741].**

*Results/Tables
• Please include the reporting for Table 1 in the results section*

**We moved and reported the participants’ characteristics and Table 1 in the results section.**

*• Table 2 seems like the information does not need to be in a table but could go in the text as bullet points*

**We eliminated Table 2 and now include the interview guide in S1 file.**

*• Table 3 seems more like a figure than a table?*

**We reformatted Table 3 (now Table 2) so readers can easily follow the themes and subthemes relevant to the objectives of the study.**

*• Line 130: please briefly describe the transcription QA process*

**We added a brief description for the transcription QA process.**
 *• Table 3, under barrier to cessation please is the lack of restriction for SLT use is in contrast to restrictions on combustible tobacco use, if so please make this explicit.*

**We clarified and changed “lack of restriction for SLT use” to “lack of enforcement of existing tobacco policies”.**
 *• Line 22: ‘personal barriers’ are called ‘Intrapersonal barriers’ on line 224, please make these terms consistent*

**We corrected the word “personal barriers” to “interpersonal barriers”.**
 *Discussion
The concluding remarks include (staring line 456) “This approach would be particularly useful for fire service, where traditions and culture can be leveraged to enhance intervention acceptance and effectiveness.” is a central point, but please introduce this concept earlier in the discussion (page 20?) For the authors consideration: Might another possible intervention approach that capitalizes on fire house culture would be to engage the firefighters who are former SLT users as a support system for current users trying to quit?*
 **We have added sentences to introduce the concept in the beginning of the discussion. We also thank the reviewer for the suggestion on a support system. We are currently developing the intervention and using former SLT users as the support system is one of the components to assist firefighters to quit SLT.**
 *The limitation of this study that it was conducted among career firefighters requires more explanation. Career firefighters make up about 33% of the US fire service and the vast majority work in urban population centers. In contrast, volunteer firefighters (67% of the US fire service) tend to serve in smaller rural communities where the prevalence if SLT is higher. As such there is a significant research gap that should be identified. As well, in key places in the manuscript, the authors should remind the readers that these are career firefighters, this should include (but not be limited to) lines: 65, 451, 82.*

**We now emphasize that our study mainly focused on career firefighters per the reviewer’s suggestion and provided more explanations on career firefighters under the limitation of the study.**

*Other edits
• Line 86: please replace the word ‘using’ in the phrase “…a sample of current and former SLT using firefighters’ with the word ‘among’.
• Line 140: this is the first instance of the use of the acronym SCT, so please define it here
• Page 351, delete “by firefighters” (it is repetitive)
• Line 353, consider replacing “holidays” with “vacation days” as (in the US) generally ‘holiday’ implies a day off for everyone and vacation day implies an individual reward*

**We edited our manuscript per the reviewer’s suggestions.**

*6. PLOS authors have the option to publish the peer review history of their article (*[*what does this mean?*](https://journals.plos.org/plosone/s/editorial-and-peer-review-process#loc-peer-review-history)*). If published, this will include your full peer review and any attached files.


If you choose “no”, your identity will remain anonymous but your review may still be made public.*


***Do you want your identity to be public for this peer review?*** *For information about this choice, including consent withdrawal, please see our* [*Privacy Policy*](https://www.plos.org/privacy-policy)*.*

*Reviewer #1: No*

*Reviewer #2:* ***Yes:*** *Judith M Graber*

*[NOTE: If reviewer comments were submitted as an attachment file, they will be attached to this email and accessible via the submission site. Please log into your account, locate the manuscript record, and check for the action link "View Attachments". If this link does not appear, there are no attachment files.]

While revising your submission, please upload your figure files to the Preflight Analysis and Conversion Engine (PACE) digital diagnostic tool,* [*https://pacev2.apexcovantage.com/*](https://pacev2.apexcovantage.com/)*. PACE helps ensure that figures meet PLOS requirements. To use PACE, you must first register as a user. Registration is free. Then, login and navigate to the UPLOAD tab, where you will find detailed instructions on how to use the tool. If you encounter any issues or have any questions when using PACE, please email PLOS at* [*figures@plos.org*](mailto:figures@plos.org)*. Please note that Supporting Information files do not need this step.*
