# Supplementary material for: A qualitative study to assess perceptions, barriers, and motivators supporting smokeless tobacco cessation in the US fire service
Source: PLoS One. 2021 May 11;16(5):e0251128. doi: 10.1371/journal.pone.0251128 (PMC8112677; doi:10.1371/journal.pone.0251128)
Supplement: Attachment — Submitted filename: Responses to Reviewers.docx [file pone.0251128.s005.docx]

**Our responses to the editor and reviewers are in bold texts below:**

Thank you for submitting your manuscript to PLOS ONE. After careful consideration, we feel that it has merit but does not fully meet PLOS ONE’s publication criteria as it currently stands. Therefore, we invite you to submit a revised version of the manuscript that addresses the points raised during the review process.

While you satisfied Reviewer 2, Reviewer 1 continued to recommend rejection of the paper, in even stronger terms than for the original submission.  For this reason, I solicited a third review. The third reviewer was also very critical and generally agreed with Reviewer 1, but thought you should be given a chance to try and fix the paper.  That is why I am giving you another chance to revise the paper.

The criticisms are, however, quite serious and will require a lot of work to fix.

**We would like to thank the Academic Editor for the careful reading and the reviewers for their high quality and constructive review of our manuscript. We have revised and edited the manuscript in response to the extensive and insightful reviewers’ comments. We hope that a revised version of the manuscript will be considered by PLOS ONE.**

Please submit your revised manuscript by Dec 26 2020 11:59PM. If you will need more time than this to complete your revisions, please reply to this message or contact the journal office at [plosone@plos.org](mailto:plosone@plos.org). When you're ready to submit your revision, log on to <https://www.editorialmanager.com/pone/> and select the 'Submissions Needing Revision' folder to locate your manuscript file.

Please include the following items when submitting your revised manuscript:

- A rebuttal letter that responds to each point raised by the academic editor and reviewer(s). You should upload this letter as a separate file labeled 'Response to Reviewers'.
- A marked-up copy of your manuscript that highlights changes made to the original version. You should upload this as a separate file labeled 'Revised Manuscript with Track Changes'.
- An unmarked version of your revised paper without tracked changes. You should upload this as a separate file labeled 'Manuscript'.

**We now include this rebuttal letter, the marked-up copy of our manuscript with track changes indicating edits made per the reviewers’ suggestions, and the unmarked version of our revised manuscript.**

If you would like to make changes to your financial disclosure, please include your updated statement in your cover letter. Guidelines for resubmitting your figure files are available below the reviewer comments at the end of this letter.

If applicable, we recommend that you deposit your laboratory protocols in [protocols.io](http://protocols.io/) to enhance the reproducibility of your results. Protocols.io assigns your protocol its own identifier (DOI) so that it can be cited independently in the future. For instructions see: <http://journals.plos.org/plosone/s/submission-guidelines#loc-laboratory-protocols>

Kind regards,

Stanton A. Glantz
Academic Editor
PLOS ONE

[Note: HTML markup is below. Please do not edit.]

Reviewers' comments:

Reviewer's Responses to Questions

**Comments to the Author**

1. If the authors have adequately addressed your comments raised in a previous round of review and you feel that this manuscript is now acceptable for publication, you may indicate that here to bypass the “Comments to the Author” section, enter your conflict of interest statement in the “Confidential to Editor” section, and submit your "Accept" recommendation.

Reviewer #1: (No Response)

Reviewer #2: All comments have been addressed

Reviewer #3: (No Response)

2. Is the manuscript technically sound, and do the data support the conclusions?

The manuscript must describe a technically sound piece of scientific research with data that supports the conclusions. Experiments must have been conducted rigorously, with appropriate controls, replication, and sample sizes. The conclusions must be drawn appropriately based on the data presented.

Reviewer #1: No

Reviewer #2: Yes

Reviewer #3: Yes

3. Has the statistical analysis been performed appropriately and rigorously?

Reviewer #1: N/A

Reviewer #2: Yes

Reviewer #3: N/A

4. Have the authors made all data underlying the findings in their manuscript fully available?

The [PLOS Data policy](http://www.plosone.org/static/policies.action#sharing) requires authors to make all data underlying the findings described in their manuscript fully available without restriction, with rare exception (please refer to the Data Availability Statement in the manuscript PDF file). The data should be provided as part of the manuscript or its supporting information, or deposited to a public repository. For example, in addition to summary statistics, the data points behind means, medians and variance measures should be available. If there are restrictions on publicly sharing data—e.g. participant privacy or use of data from a third party—those must be specified.

Reviewer #1: No

Reviewer #2: Yes

Reviewer #3: No

5. Is the manuscript presented in an intelligible fashion and written in standard English?

PLOS ONE does not copyedit accepted manuscripts, so the language in submitted articles must be clear, correct, and unambiguous. Any typographical or grammatical errors should be corrected at revision, so please note any specific errors here.

Reviewer #1: Yes

Reviewer #2: Yes

Reviewer #3: Yes

6. Review Comments to the Author

Please use the space provided to explain your answers to the questions above. You may also include additional comments for the author, including concerns about dual publication, research ethics, or publication ethics. (Please upload your review as an attachment if it exceeds 20,000 characters)

Reviewer #1: (No Response)

Reviewer #2: The authors have reviewed each of the comments and give very satisfactory responses. Many of my suggested reviews were editorial in nature and these were all addressed. My primary content concern was about variation in prevalence of smokeless tobacco use between the regions in the United States. The authors response that of smokeless tobacco use is a core common culture in the US fire service was based on their previous qualitative work in the fire service and was adequality addressed in their revised manuscript. I was also very concerned that the manuscript was not clear that they were focused on career (vs volunteer) firefighters and they have addressed this concern.

**We thank Reviewer #2 for their suggestions and comments, which helps to strengthen our manuscript.**

Reviewer #3: The revised manuscript addressed most, but not all of the concerns raised by the reviewers in the first round. There are also some additional issues that were not raised earlier, but need to be clarified.

**We would like to thank the reviewer for their high quality and constructive reviews of our manuscript. We have revised and modified our manuscript per the reviewers’ comments and suggestions. Detailed point-by-point responses to each of the reviewers’ comments are as follow:**

The biggest issue with this paper is the inconsistency: inconsistency between the use of SCT to design the study and grounded theory approach to analyze the results; inconsistency in the use of terminology, and inconsistency between the SCT constructs and the reported results. Below, I first describe these structural inconsistencies and then detail other, more minor issues.

It seems that SCT was used to design the study (to develop the interview guide) and to guide the coding. This is fine. However, the authors specify that a grounded theory approach was used to analyze the transcripts. I believe that a grounded theory approach is incompatible with an a priori selection of a theory to guide the development of the study and the analysis of the interviews. The way I understand it, a grounded theory approach implies complete openness and allowing the data to dictate the emerging themes. This study, in contrast, was guided by the SCT from the start. (This issue was raised in the first round of reviews, and simply shifting the order of the presentation for methods does not resolve it.)

The described interview guide does not quite match the SCT structure. As the authors themselves described in the introduction, two of the main aspects of the SCT is observational learning and self-efficacy. The structure for “general domains” does not list those at all. This might stem from the use of different terminology (next point).

Throughout the paper, the terminology keeps changing, sometimes reflecting the SCT constructs (“personal, social, and environmental determinants” in the introduction) and at other times something else (“cultural, community, and personal influences” in the beginning of the discussion). In the results, the structure is completely different once again. It would be helpful to consistently use the same language to refer to the same issues.

To resolve these issues regarding theoretical inconsistency, I suggest the following approach (although other approaches might be useful): first, the SCT is described in the introduction to give the readers the overview of the theory. It should be described in such a way that would match the constructs that are used in the results. It could be mentioned that SCT was used as a loose theoretical guidance only in that it indicated the broad domains (intrapersonal, environmental, etc.). Second, the description on the data analysis in the methods should specify how SCT was used to guide the analysis and whether the themes were derived deductively or inductively. If grounded theory is mentioned, it needs to be clearly explained how it was used in a combination with an a priori theoretical framework of SCT. Throughout the paper it would be helpful to keep the structure consistent.

**We now revised our manuscript based on the reviewer’s suggestive approach. First, we revised the introduction to match the SLT constructs that are used in the results (Line numbers: 102-116). Second, we revised our data analysis section (Line numbers: 161-182) to reflect on the approaches we used for data analysis and provided the description of how SCT was used to guide the analysis. Third, we now used the same language and terminology of SCT constructs throughout the manuscript. Lastly, we rearranged and presented our results according to themes and subthemes.**

In the discussion, it would be worth addressing the following issues:

Factors influencing SLT use comprise two separate categories – factors that led to initiation of the SLT use (use for the first time) and factors that make firefighters use SLT on a regular basis. Furthermore, the barriers to quitting and factors influencing SLT seem to be very similar – do they work differently as barriers vs. facilitators? For example, “social influences” under “Factors influencing SLT use” are very similar to “social and community networks” under “barriers to cessation”. Why are they called differently when they are essentially the same?

**We provided the definitions of these two categories: determinants of SLT use: “Determinants of SLT use referred to the reason and factors that influence firefighters for using SLT” (Lines number: 199-201) and barriers to cessation: “Barriers were defined as factors that made SLT cessation difficult or prevented a firefighter from quitting SLT” (Line numbers: 292-293) to avoid confusion.**

**We also arranged the findings to match our major themes per the reviewer’s suggestion. Now the subtheme “social and community networks” under “barrier to cessation” is renamed to “acceptability of SLT use in the fire service” and placed under “determinant of SLT use” (Line numbers 258-277).**

Minor issues

It is unclear if all transcripts were coded by two coders and a subset (n=5) by three coders, or if all transcripts were coded by one coder and a subset (n=5) by two coders. If the latter, then it is unclear how the discrepancies were identified and reconciled in the transcripts that were coded only by one coder.

**We now clarified and revised the sentence that two coders independently coded all 23 transcripts and the third researcher coded a subset of the transcripts (n = 5).**

**The new sentence is “Two primary coders independently coded all 23 transcripts, compared their analyses, and discussed any discrepancies until consensus was reached. Coding was regularly reviewed and verified by the third researcher to identify and incorporate emergent themes and for data validity. In addition, the third researcher conducted independent coding of a subset of data (n = 5) to ensure that the final coding scheme had adequate reliability.” (Line numbers: 177-182).**

It should be specified explicitly somewhere that all participants were men (and why women were not included – because of low SLT rates among women).

**We added the sentence indicated that women firefighters were not included in the study due to the very low SLT use prevalence compared with men in the fire service: “In addition, women firefighters were not included in the study due to the very low SLT use prevalence compared with men in the fire service (0.5%; [31]).” (Line numbers: 133-135).**

**We also reported in the result section that all participants were all men (Line number: 184).**

Were firefighters who also smoked excluded? (I.e., were these exclusive SLT users or dual users of SLT and cigarettes?) Among former SLT users, were any using other tobacco products? It would be helpful to report this information.

**We revised and clarified the definition of current and former SLT users in the method section. The revision sentence was changed to “current users - those who reported exclusively used SLT on multiple occasions within the past month; and former users - those who reported using SLT regularity in the past, but not currently using SLT or any tobacco products.” (Line numbers: 128-130).**

Some of the information in the results would be more appropriate for the discussion. For example, information on raising taxes on SLT in the section on external motivators belongs in the discussion.

**This information on raising taxes on SLT was moved to the discussion section (Line numbers: 510-514).**

Proofreading would be helpful: “SLT cessation programs that tailored”, “traditional, teamwork, and mission can be leveraged”

**We proofread the whole manuscript and corrected the grammatical errors.**

In the results, stress is mentioned in multiple places and it would be better to put it in one place. For example, it might not be needed in the “organizational barriers”.

**The stress subthemes were combined and moved under the “determinants of SLT use” and renamed to “coping with job stress” (Line numbers 278-290).**

“Beneficial perceptions of SLT use, such as relaxation, focus, and stress-management, appeared to be more important barriers to promoting SLT cessation among our study participants than perceived disadvantage around health issues, money, and addiction, which have reportedly been strong motivators in tobacco cessation in previous work [50].” – It is unclear how the comparative importance was evaluated from the qualitative results; in the results, each of the categories is reported separately. In order to be able to conclude about the comparative importance, it is necessary to demonstrate that in the results first, but it is unclear if such analysis was made. The way the results are presented now can be described as “descriptive statistics” – they simply describe the themes and issues that participants mentioned, without more in-depth analysis of how they interacted and how they, in combination, might drive the behavior. It is fine to do a descriptive presentation, but it is important to make sure that the discussion is based on the results and does not speculate without the evidence.

**This sentence is now removed from the manuscript since it is based on speculation, not the findings.**

From the first round of reviews, it does not seem like the following questions were sufficiently answered:

“Have non-tailored SLT cessation interventions been implemented among firefighters in the past? If so, how successful were they?” (The text states that culturally tailored interventions are nonexistent, but does not seem to address the issue of non-tailored SLT interventions).

**Unfortunately, the non-tailored SLT cessation interventions have not been implemented in the fire service. We now addressed the issue of non-tailored SLT interventions in fire service by revising the sentence to “Additionally, no non-tailored of the SLT education or treatment programs has been implemented or provided for the fire service, and no occupationally tailored SLT cessation intervention exists for firefighters” (Line numbers: 93-95), and we mentioned it again in the discussion section: “…no non-tailored or occupationally-tailored SLT education or treatment programs exist for the fire service.” (Lines numbers: 445-447).**

Regarding reporting the length of the interviews – it would be more appropriate to report the range (with the explanation that one interview had to be cut short due to a response to a fire call) and the median length, rather than the average length of the interview.

**We now reported the range and median length of the interview to explain that one interview was only 20 minutes due to fire response: “Researchers (NJ, CP, HK) carried out the semi-structured interviews, ranging from 20 to 50 minutes (median 42 minutes). Of the 23 interviewed participants, one participant responded to a fire call, resulting in only 20 minutes of the interview.” (Line numbers: 157-159).**

And a final note on the format of responses to the reviewers’ comments. The reviewers provide detailed and thoughtful comments, and it is expected that the responses would be equally detailed. It is difficult for a reviewer to evaluate this response: “We now provided additional details on the negative effects of SLT on firefighters.” The reviewer now has to go through the article and try and figure out where these “additional details” are and if they are sufficient. It would be a lot easier for a reviewer to evaluate a response if it included a quote with that new information directly in the response letter. (It might lengthen the response letter significantly, but in the end it would save reviewers a lot of time and make them happier.)

**We would like to thank the reviewer for the suggestion. We now included a quote with the new or additional information responding to reviewers’ comments. We also provided line numbers corresponding to the revised manuscript to navigate the reviewer to our responses.**

7. PLOS authors have the option to publish the peer review history of their article ([what does this mean?](https://journals.plos.org/plosone/s/editorial-and-peer-review-process#loc-peer-review-history)). If published, this will include your full peer review and any attached files.


If you choose “no”, your identity will remain anonymous but your review may still be made public.


**Do you want your identity to be public for this peer review?** For information about this choice, including consent withdrawal, please see our [Privacy Policy](https://www.plos.org/privacy-policy).

Reviewer #1: No

Reviewer #2: No

Reviewer #3: No

[NOTE: If reviewer comments were submitted as an attachment file, they will be attached to this email and accessible via the submission site. Please log into your account, locate the manuscript record, and check for the action link "View Attachments". If this link does not appear, there are no attachment files.]

While revising your submission, please upload your figure files to the Preflight Analysis and Conversion Engine (PACE) digital diagnostic tool, <https://pacev2.apexcovantage.com/>. PACE helps ensure that figures meet PLOS requirements. To use PACE, you must first register as a user. Registration is free. Then, login and navigate to the UPLOAD tab, where you will find detailed instructions on how to use the tool. If you encounter any issues or have any questions when using PACE, please email PLOS at [figures@plos.org](mailto:figures@plos.org). Please note that Supporting Information files do not need this step.
