# Supplementary material for: A qualitative study to assess perceptions, barriers, and motivators supporting smokeless tobacco cessation in the US fire service
Source: PLoS One. 2021 May 11;16(5):e0251128. doi: 10.1371/journal.pone.0251128 (PMC8112677; doi:10.1371/journal.pone.0251128)
Supplement: Attachment — Submitted filename: Responses to Reviewers.docx [file pone.0251128.s006.docx]

**Our responses to the editor and reviewers are in bold texts below:**

Thank you for submitting your manuscript to PLOS ONE. After careful consideration, we feel that it has merit but does not fully meet PLOS ONE’s publication criteria as it currently stands. Therefore, we invite you to submit a revised version of the manuscript that addresses the points raised during the review process.

**We would like to thank the Academic Editor for the careful reading and the reviewers for their high quality and constructive review of our manuscript. We have revised and edited the manuscript in response to the extensive and insightful reviewers’ comments. We hope that a revised version of the manuscript will be considered by PLOS ONE.**

Please submit your revised manuscript by May 13 2021 11:59PM. If you will need more time than this to complete your revisions, please reply to this message or contact the journal office at [plosone@plos.org](mailto:plosone@plos.org). When you're ready to submit your revision, log on to <https://www.editorialmanager.com/pone/> and select the 'Submissions Needing Revision' folder to locate your manuscript file.

Please include the following items when submitting your revised manuscript:

- A rebuttal letter that responds to each point raised by the academic editor and reviewer(s). You should upload this letter as a separate file labeled 'Response to Reviewers'.
- A marked-up copy of your manuscript that highlights changes made to the original version. You should upload this as a separate file labeled 'Revised Manuscript with Track Changes'.
- An unmarked version of your revised paper without tracked changes. You should upload this as a separate file labeled 'Manuscript'.

**We now include this rebuttal letter, the marked-up copy of our manuscript with track changes indicating edits made per the reviewers’ suggestions, and the unmarked version of our revised manuscript.**

If you would like to make changes to your financial disclosure, please include your updated statement in your cover letter. Guidelines for resubmitting your figure files are available below the reviewer comments at the end of this letter.

If applicable, we recommend that you deposit your laboratory protocols in [protocols.io](http://protocols.io/) to enhance the reproducibility of your results. Protocols.io assigns your protocol its own identifier (DOI) so that it can be cited independently in the future. For instructions see: <http://journals.plos.org/plosone/s/submission-guidelines#loc-laboratory-protocols>. Additionally, PLOS ONE offers an option for publishing peer-reviewed Lab Protocol articles, which describe protocols hosted on [protocols.io](http://protocols.io/). Read more information on sharing protocols at <https://plos.org/protocols?utm_medium=editorial-email&utm_source=authorletters&utm_campaign=protocols>.

We look forward to receiving your revised manuscript.

Kind regards,

Stanton A. Glantz
Academic Editor
PLOS ONE

Journal Requirements:

Please review your reference list to ensure that it is complete and correct. If you have cited papers that have been retracted, please include the rationale for doing so in the manuscript text, or remove these references and replace them with relevant current references. Any changes to the reference list should be mentioned in the rebuttal letter that accompanies your revised manuscript. If you need to cite a retracted article, indicate the article’s retracted status in the References list and also include a citation and full reference for the retraction notice.


[Note: HTML markup is below. Please do not edit.]

Reviewers' comments:

Reviewer's Responses to Questions

**Comments to the Author**

1. If the authors have adequately addressed your comments raised in a previous round of review and you feel that this manuscript is now acceptable for publication, you may indicate that here to bypass the “Comments to the Author” section, enter your conflict of interest statement in the “Confidential to Editor” section, and submit your "Accept" recommendation.

Reviewer #3: (No Response)

2. Is the manuscript technically sound, and do the data support the conclusions?

The manuscript must describe a technically sound piece of scientific research with data that supports the conclusions. Experiments must have been conducted rigorously, with appropriate controls, replication, and sample sizes. The conclusions must be drawn appropriately based on the data presented.

Reviewer #3: Yes

3. Has the statistical analysis been performed appropriately and rigorously?

Reviewer #3: N/A

4. Have the authors made all data underlying the findings in their manuscript fully available?

The [PLOS Data policy](http://www.plosone.org/static/policies.action#sharing) requires authors to make all data underlying the findings described in their manuscript fully available without restriction, with rare exception (please refer to the Data Availability Statement in the manuscript PDF file). The data should be provided as part of the manuscript or its supporting information, or deposited to a public repository. For example, in addition to summary statistics, the data points behind means, medians and variance measures should be available. If there are restrictions on publicly sharing data—e.g. participant privacy or use of data from a third party—those must be specified.

Reviewer #3: Yes

5. Is the manuscript presented in an intelligible fashion and written in standard English?

PLOS ONE does not copyedit accepted manuscripts, so the language in submitted articles must be clear, correct, and unambiguous. Any typographical or grammatical errors should be corrected at revision, so please note any specific errors here.

Reviewer #3: Yes

6. Review Comments to the Author

Please use the space provided to explain your answers to the questions above. You may also include additional comments for the author, including concerns about dual publication, research ethics, or publication ethics. (Please upload your review as an attachment if it exceeds 20,000 characters)

Reviewer #3: The revisions mostly sufficiently addressed the issues raised in the previous round of review. The issue with the lack SCT constructs and the presentation of the results has been mostly resolved. There is one issue with the lack of clarity regarding the conceptual distinction between determinants and barriers, described below.

**We would like to thank the reviewer for their high quality and constructive reviews of our manuscript. We have revised and modified our manuscript per the reviewers’ comments and suggestions. Detailed point-by-point responses to each of the reviewers’ comments are as follow:**

The section “Acceptability of SLT use in the fire service” is in the section on “Determinants of SLT use”, but it talks about barriers to cessation: “The high prevalence and social acceptability of SLT within fire service communities were cited as making quit attempts challenging. Some participants expressed that offering SLT or accepting SLT from other firefighters was the reason for their failure to quit, i.e., it was common for them to offer or receive other firefighters SLT as a sign of a close relationship or as being a team member or as a way to show “camaraderie”.” It would be better to rewrite these sentences to focus on social norms and widespread use as leading to use, rather than preventing the cessation.

**We revised the “Acceptability of SLT use in the fire service” section to focus on social norms and widespread of SLT use. (Line numbers: 257-281).**

The same issue is with the next section – “coping with job stress”: “The nature of firefighting tasks and the stressful context of the job were frequently mentioned and presented as unique barriers to SLT cessation for firefighters”. If it’s in the “Determinants of SLT use” section, it should be discussed as a determinant.

**We also revised the “Coping with job stress” section to be more related to the determinants of SLT use. (Line numbers: 282-295).**

Related to the two examples above, it seems that in the previous version they were in the “barriers to cessation” section and then were simply moved from the “barriers” to “determinants” section. This goes back to my comment from the previous round – how are “determinants” and “barriers” conceptually different? Clearly, some things that make SLT use attractive in the first place make it harder to quit too (e.g., widespread use by peers). It might be worthwhile to indicate that some of the “determinants” were discussed as both “determinants” and “barriers” and that the section “barriers to cessation” explicitly deals with only those issues that focus on preventing cessation (or something like that).

**We thank the reviewer for their suggestions. We now revised the discussion section to reflect on our findings.**

In addition to this issue, there are some additional minor issues with language and focus, detailed next.
“Additionally, no non-tailored of the SLT education or treatment programs have been implemented or provided for the fire service, and no occupationally tailored SLT cessation intervention exists for firefighters.” – suggest changing to: “Additionally, no SLT education or treatment programs have been implemented for the fire service, and no occupationally tailored SLT cessation interventions exist for firefighters.”

**We revised this sentence per the reviewer’s suggestion (Line numbers: 93-95).**

“current users - those who reported exclusively used SLT on multiple occasions” – should be “current users - those who reported exclusively using SLT on multiple occasions”

**We revised this sentence per the reviewer’s suggestion (Line numbers: 127-128).**

Spell out “EAP” the first time it is used.

**We now provided the full term of the EAP when it first mentioned (Line number: 349).**

For Table 1, it might be more informative to provide the min and max of age years and time in service (years). Since there were two groups (current users and former users), it might be useful to provide separate columns for them. This way Table 1 will give a lot more information without repeating what is already said in the text.

**We edited Table 1 and provided the demographic characteristics for both former- and current users in separate columns. We also provided the range (min-max) of age (years) and time in service (years).**

This quote seems to belong in the section on “lack of support from health and other service providers” rather than in the section on “lack of enforcement of existing tobacco policies” where it currently is: “They don't really have anything on the market for smokeless. Everything is geared towards smokers. Getting a nicotine patch or something like that, you get the nicotine, but you don't get the feeling of it in your lip. And I think that is a big part of it.” (P32,
445 user)”

**We moved this quote to the appropriate section, “lack of support from health and other service providers” (Line numbers: 357-360).**

The paper needs to be carefully proofread to make sure the changes that were made correspond to the language left over from the earlier version. For example, the discussion lists 3 barriers for SLT cessation (“Given that this qualitative study identified three primary barriers for SLT cessation in fire service personnel”), but the section on barriers has either 2 or 4 (depending on the level).

**We proofread the whole manuscript and corrected the grammatical errors.**

Finally, in the discussion, there is a lot of general talk about how the study can inform the intervention and how a targeted intervention would be better. This information appears at least three times, and all those can be condensed. Recommendations for an intervention are likewise dispersed throughout the discussion. It would be better to clearly state – based on the findings, what an intervention (or series of interventions) for career firefighters should look like?

**We would like to thank the reviewer for the suggestion. We now revised the discussion section to be clearer and more succinct.**

7. PLOS authors have the option to publish the peer review history of their article ([what does this mean?](https://journals.plos.org/plosone/s/editorial-and-peer-review-process#loc-peer-review-history)). If published, this will include your full peer review and any attached files.


If you choose “no”, your identity will remain anonymous but your review may still be made public.


**Do you want your identity to be public for this peer review?** For information about this choice, including consent withdrawal, please see our [Privacy Policy](https://www.plos.org/privacy-policy).

Reviewer #3: No

[NOTE: If reviewer comments were submitted as an attachment file, they will be attached to this email and accessible via the submission site. Please log into your account, locate the manuscript record, and check for the action link "View Attachments". If this link does not appear, there are no attachment files.]

While revising your submission, please upload your figure files to the Preflight Analysis and Conversion Engine (PACE) digital diagnostic tool, <https://pacev2.apexcovantage.com/>. PACE helps ensure that figures meet PLOS requirements. To use PACE, you must first register as a user. Registration is free. Then, login and navigate to the UPLOAD tab, where you will find detailed instructions on how to use the tool. If you encounter any issues or have any questions when using PACE, please email PLOS at [figures@plos.org](mailto:figures@plos.org). Please note that Supporting Information files do not need this step.
